# The Physicochemical, Microbiological, and Structural Changes in Beef Are Dependent on the Ultrasound System, Time, and One-Side Exposition

**DOI:** 10.3390/molecules27020541

**Published:** 2022-01-15

**Authors:** Luis M. Carrillo-Lopez, Bianka Y. Cruz-Garibaldi, Mariana Huerta-Jimenez, Ivan A. Garcia-Galicia, Alma D. Alarcon-Rojo

**Affiliations:** 1 National Council of Science and Technology, Av. Insurgentes Sur 1582, Col. Crédito Constructor, Del. Benito Juárez, Ciudad de México 03940, Mexico; mhuertaj@uach.mx; 2 Faculty of Animal Science and Ecology, Autonomous University of Chihuahua, Perif. Francisco R. Almada km 1, Chihuahua 31453, Mexico; p334599@uach.mx (B.Y.C.-G.); aalarcon@uach.mx (A.D.A.-R.)

**Keywords:** beef, high-intensity ultrasound, meat quality, microstructure, bacterial control

## Abstract

The effect of high-intensity ultrasound (HIU) system (bath, 37 kHz and 90 W/cm^2^; or probe, 24 kHz and 400 W) and application time (25 or 50 min, one-side exposition) on the properties of bovine *Longissimus lumborum* after 7 d of storage at 4 °C was studied. The bath system significantly increased the lightness of the muscle, while other color parameters (a*, b*, hue, and chroma) were not different from the control. The water holding capacity and shear force decreased significantly (3.1–5% and 0.59–0.72 kgf, respectively) in sonicated meat independently of the system, favoring the tenderization of the muscle after storage. Microstructural changes observed in the HIU-exposed surface provided evidence of a higher area of interfibrillar spaces (1813 vs. 705 µm^2^ in the control), producing tenderization of the muscle, compared with the control. HIU significantly increased counts of total aerobic and coliform bacteria, especially after 50 min of ultrasonication. HIU also increased lactic acid bacterial counts in the bath system. Single-sided muscle exposition to ultrasound may produce sufficient significant changes in muscle properties, which could decrease long treatment times that would be needed for the exposition of both sides. HIU in bath systems increases tenderness by modifying meat ultrastructure, with no significant changes in physicochemical parameters. Nevertheless, microbiological quality may need to be considered during the process due to a slight increase in bacterial counts.

## 1. Introduction

High intensity and low-frequency ultrasound (HIU. F = 20–100 kHz, I = >1 W/cm^2^) may modify the physicochemical and structural properties of foods [1]. Its application can be direct, coupled to a device (probe), or in an ultrasonic bath (immersion) [2]. Beneficial effects of HIU applied on meat have been reported in various processes such as marinating, freezing, drying, emulsification, and storage. Yet, industrial scale-up is limited due to the need for further study and analysis of desirable and undesirable changes produced in foods. On this aspect, several authors have reported changes in quality attributes of the meat, which are dependent on the experimental conditions and parameters of HIU [2,3,4,5].

Regarding the ultrasonication of meat, there are intrinsic characteristics of the sample (size, post-mortem time, type of muscle, packaging, atmosphere, etc.) that should be considered as part of the experimental design, additionally to equipment conditions (intensity, frequency, temperature, time, pulse, amplitude, probe size, etc.), in order to standardize the process for industrial applications [6]. The probe system has been used mainly in marinating processes [7,8,9], and the enhancement in enzyme activity in meat, producing structural changes and denaturation of proteins [10,11]. These changes increase lipid and protein oxidation, proteolysis, water holding capacity, tenderization, and mass transfer. Many studies have considered the ultrasound-probe application to fresh meat during storage and aging [12,13]. However, recent research has focused on marinating processes and myofibrillar protein solutions because the irradiation distance is limited to a specific area of the tip of the sonotrode [14]. Although probe-type devices have a localized and uniform input of ultrasonic energy producing higher intensity and efficiency during sonication, the bath system may be more practical in the meat industry, considering the use of large tanks that allow fast and efficient placement of carcasses, as well as primary and secondary cuts [1].

Fresh muscle tissue is a very complex matrix; hence, the need for research in myofibrillar protein suspensions has recently been emphasized [15]. However, basic research is needed to consider the application of ultrasound as an assisted technology in the meat industry for primary and secondary commercial cuts. The bath system produces low energy, and the cavitation is uncontrolled distributed in the tank [16], but the ultrasonic energy is not limited to a reduced area. More research is needed to standardize the parameters during the ultrasonic treatment since the results of the research are still highly variable with positive (tenderization and higher water holding capacity) and negative (toughening and release of water) effects reported. [1]. In this regard, Wang et al. [12] found that the ultrasonication of bovine *Semitendinosus* (8 × 7 × 2.5 cm) in a probe system (20 kHz, 25 W/cm^2^, 20–40 min) produced tenderization and structural changes of proteins during maturation (3–7 d). Contrarily, Alves et al. [13] reported that the treatment of bovine *Semitendinosus* with ultrasound in a probe system (20 kHz, 750 W, 26.5 W/cm^2^) or bath (45 kHz, 500 W, 1.8 W) for 0, 60, 120, or 240 s, reduced shear stress only after ultrasonication, but this effect was not maintained during storage (16 d at 7 °C). They also did not observe changes in lipid oxidation or microbial flora, so they suggested that ultrasonication should also be applied during storage. Consequently, the time of ultrasonication is important to produce permanent structural changes during meat storage. The application of ultrasound in the bath system has also shown results dependent on the experimental conditions. While Peña-Gonzalez et al. [17] reported an increase in lipid oxidation and a reduction in the toughness of bovine *Longissimus dorsi* treated with ultrasound (40 kHz, 11 W/cm^2^, 60 min) after 14 d of storage at 4 °C, Chang et al. [18], stated that the reduction in the shear stress of bovine *Semitendinosus* is significant only when long HIU times are used (> 30 min, 40 kHz, 1500 W). HIU produces cavitation, which contributes to the antimicrobial effect and longer shelf life of food items. Cavitation generates shock waves that induce damage to cell membranes and tenderize meat, either by the physical weakening of muscle structures or by the activation of enzyme systems [19]. The effect of HIU is not homogeneous in muscle tissue because ultrasonic baths do not generate a homogeneous pattern since the acoustic emitters have an overlap of intensities of the same type of emission [1,20]. The presence of packaging in meat also influences the heterogeneous effect that acoustic cavitation has on tissue. Additionally, in several studies the muscle sample was turned to promote direct contact with the ultrasonic waves on both sides, aiming for more homogeneous and efficient treatment. Nevertheless, this method leads to a significant increase in treatment time. In this sense, Caraveo et al. [21] ultrasonicated bovine m. *Semitendinosus* for 60 and 90 min. Furthermore, Barekat and Soltanizadeh [10] used proteolytic enzymes to promote tenderization of m. *Longissimus lumborum* by ultrasonication for shorter periods of 20 min. 

Under this scenario, this study evaluated the effect of the ultrasound system (bath or probe) and the treatment time (25 and 50 min) on the physicochemical, microbiological, and microstructural characteristics of bovine *Longissimus lumborum* stored for 7 days at 4 °C, prioritizing the effect of one-side exposition to the ultrasonication. 

## 2. Results and Discussion

### 2.1. CIE L*a*b* Color

Significant differences in lightness (L*) of *Longissimus lumborum* (Table 1) due to the effect of the HIU system factor (*p* = 0.0239) were observed. The muscles ultrasonicated in the bath system had a significant increase in lightness, compared with the controls. Ultrasonication time had no effect on muscle lightness (*p* = 0.8397), and the interaction between factors was not significant either (*p* = 0.9324). Since the L* coordinate is directly related to the brightness or ability to reflect color, the increase in L* in the bath system is the best treatment to increase the visual appearance of the meat. Regarding the color parameters a*, b*, chroma, and hue, no statistical differences were found for the evaluated factors (*p* > 0.05) or in the interaction between them (*p* > 0.05). These results are positive because they indicate that HIU treatment does not modify the redness (a*) or the tone (H*) and saturation (C*) (brightness, opacity) of the muscle (Table 1). Jayasooriya et al. [22] obtained similar results when using *Longissimus* (*lumborum* and *thoracis*) and *Semitendinosus* muscles 60 × 40 × 20 mm samples treated with 12 W /cm^2^ and 24 kHz for 240 s and later aged for 8.5 d were not affected in their a* and b* parameters. However, after aging, they observed a significant increase (*p* = 0.0005) in these parameters. Diaz-Almanza et al. [23] also obtained similar results to ours. They reported a significant increase in L* (*p* < 0.05) in *Longissimus lumborum* 2.5 cm thick steaks. After the HIU application in a bath system (37 kHz, 90 W/cm^2^) to 0, 10, 20, and 40 min, L* increased significantly as the HIU application time increased. On the other hand, Caraveo et al. [21] found a significant difference (*p* < 0.05) between the control group (0 min) and the ultrasonicated samples (40 kHz, 11 W/cm²). The L* increased in the HIU treated samples, without showing a significant difference between exposure times (60 and 90 min). Furthermore, L* tends to increase until day 6 of storage, but on day 10, L* decreased to similar values on day 0. Regarding a* and b* coordinates, contrary to the results of this study, Caraveo et al. [21] showed that the control meat presented significantly higher values (*p* < 0.05) than the samples treated with HIU until day 6 of storage. On day 8 of storage, there was a non-significant increase, to remain constant until day 10.

### 2.2. PH

**Figure 1:**
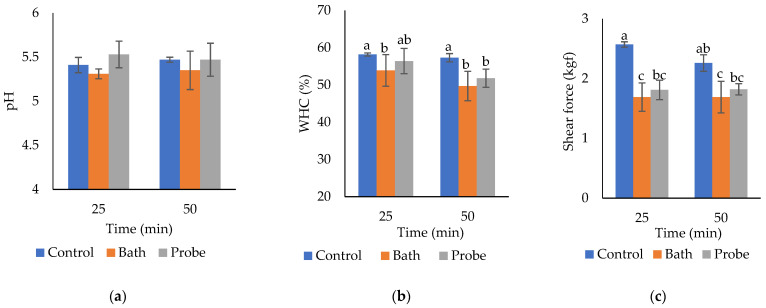
Effect of the combination of HIU system and HIU time factors on pH (**a**), water holding capacity (WHC, %) (**b**), and shear force (kgf) (**c**) of bovine *Longissimus lumborum*. ^a,b,c^ Different letters in the columns within the same graph indicate significant differences between treatments (*p* < 0.05).

The pH values were within normal values for bovine *Longissimus lumborum*. No significant differences were found in the pH of *Longissimus lumborum* by effect of the HIU system (*p* = 0.0724, Table 2), treatment time (*p* = 0.7665, Table 2), nor by the interaction of factors (*p* = 0.6155, Figure 1a). The bath system tended to reduce the pH of the muscle (from 5.44 in the controls to 5.33 in the bath system), while in the probe system the pH increased slightly (up to 5.53). However, the differences were not significant. Therefore, the HIU treatment did not produce significant changes in the pH of m. *Longissimus lumborum*. According to Jayasooriya et al. [22], the pH of the muscles of *Longissimus lumborum* (LL), *Longissimus thoracis* (LLT), and *Semitendinosus* (ST) remained in a range of 5.37–5.71 after treatment with HIU (24 kHz, 12 W/cm^2^). Even though after 8.5 d of aging, the pH increased, the differences were not significant. Got et al. [19] also did not report significant changes in the final pH of m. *Semimembranosus* (50 g) by effect of ultrasound treatment (2.6 MHz, 10 W/cm^2^, 2 × 15 s) before (day 0, pH 6.2) or after rigor (day 1, pH 5.4). Diaz-Almanza et al. [23] found similar results to ours. In this case, they treated 2.5 cm thick sections in a bath system (90 W/cm^2^, 37 kHz) with HIU. Contrarily, Wang et al. [12] reported that the pH of meat treated with HIU (samples of 80 × 70 × 25 mm, m. *Semitendinosus*) in an ultrasonic probe system (25 W/cm^2^) for 20 and 40 min was significantly higher (*p* < 0.05) immediately after treatment. However, they did not observe significant changes after aging. The increase in pH in muscle may be due to the release of ions in the muscle cell or to changes in the protein structure [1]. Caraveo et al. [21] reported that HIU application (11 W/cm^2^, 40 kHz) in sections of 1.27 cm thick during 60 and 90 min produced a significant decrease in the muscle pH (*p* < 0.05). These researchers reported pH ranges between 5.3 and 5.6.

### 2.3. Water Holding Capacity (WHC)

The results showed a significant decrease in the WHC of *Longissimus lumborum* treated with HIU (*p* = 0.0142), regardless of the system (probe or bath, Table 2). The decrease in WHC is a negative effect on quality since the muscle has less capacity to retain water, significantly decreasing juiciness and performance. Increasing the sonication time (from 25 to 50 min) significantly decreased the WHC of bovine *Longissimus lumborum* (*p* = 0.0389) (Table 2). The combination HIU system * time was significant (*p* = 0.026, Figure 1b). Hence, the WHC in each system (control without US, bath, or probe) presented significant changes when increasing the ultrasonication time (from 25 to 50 min) (Figure 1b). Control without HIU (25 or 50 min) was the best combination for WHC, while treatments with 50 min in bath and probe significantly reduced the WHC in the muscle. The effect of HIU on muscle WHC is variable, depending on the experimental conditions and the characteristics of the sample, which makes the comparison among studies very difficult. Zou et al. [24] reported a significant increase in WHC and a decrease in cooking losses of cooked spiced beef (80, 100, and 120 min) with the application of HIU (0, 400, 600, 800, and 1000 W, and 20 kHz). This was attributed to the loss of pressure and free water content and an improvement in the immobilized water content. According to this research, the application of HIU generates cell disruption that increases the intermyofibrillar spaces, causing the opening of channels that retain water molecules. The study carried out by Kang et al. [7] also reported a significant increase in the WHC of HIU-assisted meat marination (150 and 300 W; 30 and 120 min). However, in fresh meat, McDonnell et al. [25] did not observe a significant difference in the WHC (*p* > 0.05) of pork *Longissimus thoracis* et *lumborum* ultrasonicated (19 W/cm^2^) for 10, 25, and 45 min.

### 2.4. Shear Force

The results showed a significant decrease in the shear force of the *Longissimus lumborum* muscle due to the effect of HIU (*p* < 0.0001), regardless of the system (probe or bath) (Table 2). The decrease in muscle toughness due to the effect of HIU treatment constitutes a novel finding, which makes this emerging technology promising under the experimental conditions of this study. Several researchers have reported an increase in meat tenderness due to the effect of ultrasonication [17,18,26,27,28]. Regarding the effect of HIU time (control, bath, and probe), there was not a significant difference in the shear force of *Longissimus lumborum* when using 25 or 50 min (*p* = 0.2404) (Figure 1b). The combination of the HIU system * HIU time was significant (*p* = 0.0002). Figure 1c shows that HIU increases muscle tenderness in the bath or probe system, regardless of the treatment time. Hence, 25 min of ultrasonication is sufficient to produce tenderization in *L. lumborum* slices of 1.25 cm. Consequently, increasing HIU time above 25 min has no additional effect on *Longissimus lumborum* shear force. Similar results were reported by Peña-Gonzalez et al. [17], who observed significant changes in the shear force (*p* < 0.0001) of *Longissimus lumborum* (13 × 9 × 2.5 cm samples) treated with HIU for 60 min (30 min/side), compared with their control group. Similarly, Diaz-Almanza et al. [23] showed that the shear force decreased significantly when HIU exposure was longer. However, the shear force values obtained by these researchers were higher than those observed in the present study. This effect could be attributed to the use of vacuum packing, which could attenuate the contact of acoustic cavitation with the sample [29] when using bags of 70 μm thickness. In this research, we demonstrated that 25 min of ultrasonication is sufficient to increase the tenderness of *Longissimus lumborum* after 7 d of storage at 4 °C, without the need for ultrasonication for 50 min.

In Table 3, Pearson correlations of shear force and other variables are shown. Shear force or toughness is one of the quality traits more important for consumers and more dependent on other parameters such as WHC, lightness, and chroma. This makes the tenderness of the meat a variable extremely affected by intrinsic and extrinsic factors along the production process. Hence, since high-intensity ultrasonication is able to reduce the shear force of meat, the technology has been considered highly promissory for industrial applications [1]. 

In Table 3, other quality characteristics such as redness (a*) and chroma (C*) results are related to shear force in beef. Lightness (L*), which is related to the superficial water of meat and the water activity in the tissue [1], was also negatively correlated to shear force. 

### 2.5. Microbiological Evaluations

**Figure 2:**
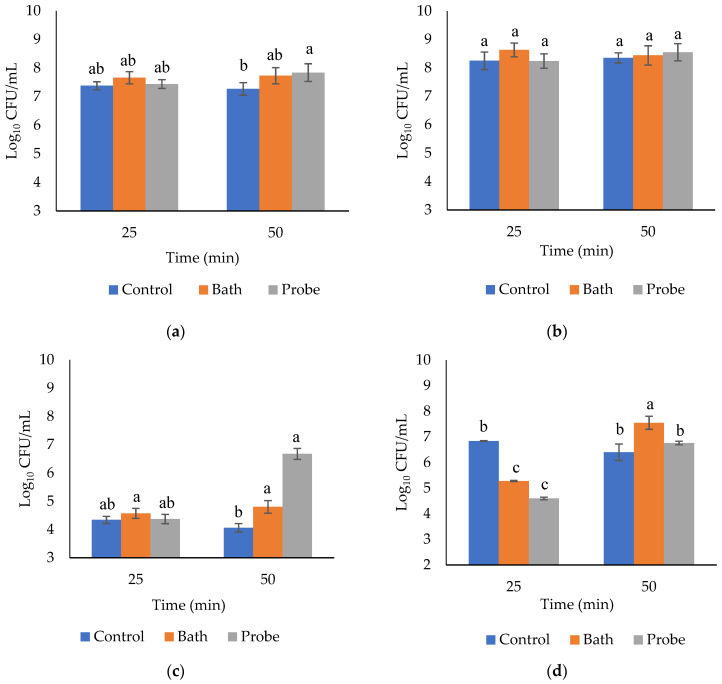
Effect of the combination of the HIU system and HIU time on the count of mesophiles (**a**), psychrophiles (**b**), lactic acid bacteria (**c**), and total coliform bacteria (**d**) of bovine *Longissimus lumborum*. ^a,b,c^ Different letters in the columns within the same graph indicate significant differences between treatments (*p* < 0.05).

The microbiological analysis showed significant differences in the counts of total aerobic flora in m. *Longissimus lumborum* due to the effect of the HIU system (*p* = 0.0089). HIU significantly increased the counts of mesophiles, from 7.32 log_10_ CFU/mL in the control (without HIU) to 7.7 and 7.64 in the bath and probe system, respectively (Table 4). The treatment time (20 or 50 min) had no significant effect on the counts of mesophiles (*p* = 0.2139, Table 3), while the interaction between factors was significant (*p* = 0.0154, Figure 2a). There was not a significant increase in mesophiles in the probe system when the ultrasonication time increased from 25 to 50 min (Figure 2a). Therefore, the least effective combination for decontamination was the probe system for 50 min, which actually increased mesophiles in meat after storage. Therefore, ultrasound alone is not an appropriate technology for the control of mesophilic aerobic bacteria. Piñon et al. [30] ultrasonicated (9.6 W/cm^2^, 40 kHz) chicken breasts for 0, 30, and 50 min. They found a significant decrease in the count of mesophilic bacteria immediately after the HIU application (≅5 log_10_ CFU/mL). However, these results were not permanent, since at 7 d after storage there was a significant increase (*p* < 0.0001, ≅7.4 log_10_ CFU/mL) in mesophiles. Those results are similar to the obtained in the present study after 7 d storage at 4 °C. Díaz-Almanza et al. [23] also obtained similar results in *Longissimus lumborum* treated under an ultrasonic bath system (90 W/cm^2^, 37 kHz) for 0, 10, 20, and 40 min. Their mesophiles counts were developed immediately after the ultrasonication, which is the reason why they were lower than the counts in the present study after 7 d of storage at 4 °C. The count of mesophiles decreased significantly after 10 min of HIU application (from ≅4.4 log_10_ CFU/mL to ≅4 log_10_ CFU/mL). However, a significant increase (*p* < 0.05) in mesophiles was observed after 20 min of HIU (≅4.2 log_10_ CFU/mL), and another non-significant increase at 40 min of ultrasonication (≅4.25 log_10_ CFU/mL). Mesophilic microorganisms are relevant in the food industry when the storage temperature rises to 15 °C. Mukhopadhyay et al. [31] suggested that transient cavitation (short and violent bursts) in low-frequency ranges may be more effective than stable cavitation (less violent with vibrations in gaseous bodies) for microbial inactivation. According to Mukhopadhyay et al. [31], with a higher number of bubbles and a longer duration of the cavitation intensity, a higher inactivation of microorganisms may be achieved. However, under the experimental conditions of this study, the HIU should be accompanied by other techniques such as the use of temperature (thermosonication) or pressure (manosonication), to be effective for microbial reduction.

Regarding bacteria growing under refrigeration conditions, no significant differences of the psychrophiles counts in bovine *Longissimus lumborum* due to the effect of the HIU system (Table 4, *p* = 0.2661), treatment time (Table 4, *p* = 8.53), or their interaction (Figure 2b, *p* = 0.309) were found. It is hypothesized that vacuum packing constitutes a barrier that prevents the efficient effect of the cavitation, which inhibits the decrease in microbial load. Thus, vacuum packing was able to attenuate direct contact with ultrasonic waves in both ultrasonic systems. According to Piñon et al. [30], under conditions of vacuum packaging and adequate refrigeration, the counts of psychrophile bacteria were not significantly different during storage. However, consistent with the present study, they reported a decrease in the count of psychrophiles immediately after HIU treatment (850, 20, and 40 kHz) in 150 g portions of chicken meat. However, they did not find a significant difference (*p* = 0.7619) at 7 d of storage. Dolatowski and Stasiak [32] also reported no significant difference in the counts of psychrophiles in meat, because of ultrasonication (25 kHz and 2 W/cm^2^) before and after the injection of brine under refrigerated conditions. Contrarily, Carrillo-Lopez et al. [6] observed that treatment with HIU (90 W/cm^2^, 20 and 40 min) in 2.5 cm slices of *Longissimus lumborum* significantly increased the counts of psychrophilic bacteria (*p* < 0.0001) after 7 d of storage under refrigerated conditions. In a study carried out by Caraveo et al. [21], the ultrasound treatment (11 W/cm^2^, 40 kHz, 60 and 90 min) in cuts of (1.27 cm) m. *Semitendinosus* produced significant differences in psychrophiles counts, compared with the control group. This showed that the exposure time to HIU is relevant in microbial inactivation. 

HIU (probe or bath) significantly increased the counts of coliform bacteria in m. *Longissimus lumborum*. However, no differences were observed due to treatment time (*p* = 0.3251, Table 4). The least effective treatment for coliforms reduction was the probe system 50 min since they significantly increase the coliform counts in the muscle (*p* = 0.0034, Figure 2c) in comparison with the control. The application of HIU by itself does not eliminate or inactivate pathogens especially when using a probe system. Therefore, an assisted technology is necessary to allow efficient control of coliform bacteria in *L. lumborum*. In this regard, several studies have focused on the use of temperature in combination with HIU to achieve efficient bacterial control in food. The application of ultrasound in combination with heat treatment (53 ± 1 °C) reduced loads of *Campylobacter jejuni*, *Enterobacteriaceae,* and total counts (total viable count) [33]. Therefore, these bacteria were sensitive to high-intensity thermosonication. Diaz-Almanza et al. [23] reported that the count of coliform bacteria decreased significantly (*p* < 0.05) after 10 min of ultrasonication, but later, there was a significant increase (*p* < 0.05) as the HIU time increased. These researchers used meat without packaging, so there was direct contact of the muscle with the ultrasonic waves. This probably reduced the microbial load significantly, compared with the results obtained in the present study, in which the packaging could obstruct or attenuate the effect of the HIU [29] on the *L. dorsi* cuts. In another study, Carrillo-Lopez et al. [6] observed a significant increase in coliform counts after treatment with HIU (90 W/cm^2^, 20 and 40 min) in 2.54 cm sections of *L. dorsi* stored at 4 °C for 7 d. In contrast, Caraveo et al. [21] observed that coliform bacteria were significantly affected by ultrasonication (11 W/cm^2^, 40 kHz, 60 and 90 min) in 1.27 cm thick sections stored for 1, 6, and 10 d. It should be considered that those authors used treatment times above 50 min.

Regarding lactic acid bacteria (LAB), the results showed significant differences because of HIU system (*p* < 0.0001, Table 4), treatment time (*p* < 0.0001, Table 4), and interaction of factors (*p* < 0.0001, Figure 2d). The increase in the growth of LAB constitutes a benefit during meat storage since it is well known that this group of bacteria has an antagonistic effect against pathogenic bacteria such as *Escherichia coli*, *Listeria*, *Staphylococcus*, *Streptococcus*, *Salmonella*, and *Pseudomonas* [30,34]. Times of 50 min in bath system seems to be the ideal combination for the increase in this group of bacteria, while the 25 min treatments tend to significantly decrease LAB counts after the storage period at 4 °C (Figure 2d). Ultrasonication in the bath system (25 kHz, 500 W) increased LAB as ultrasonication time increased from 3 to 9 min [35]. It has been proposed that ultrasonication promotes bacterial growth by microbial dispersion and the formation of temporary pores in the bacterial membrane, which allows the release and transport of nutrients and oxygen, improving cellular viability [36,37]. These effects are temporal, and they are reduced within a few hours [38]; hence, it is possible that during storage, the nutrients of macromolecules breakup by ultrasonic cavitation are available for bacterial growth. Theoretically, an increase in LAB was expected because anaerobic conditions were favored during vacuum packing, which is ideal for the development of this group of bacteria. However, our results are opposite to those obtained in the study by Piñon et al. [30], who reported that during the treatment of chicken breasts (portions of 150 g, vacuum packed, 30 and 60 min with HIU at 40 kHz and 9.6 W/cm^2^), no significant differences were found in the LAB counts (*p* = 0.2207).

### 2.6. Microstructural Studies

**Figure 3:**
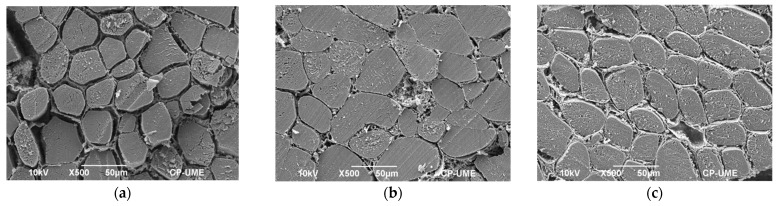

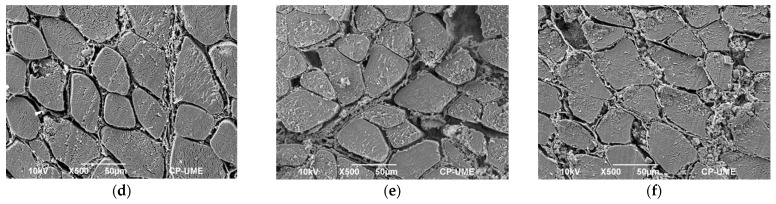
Cross-sectional scanning electron micrographs of bovine *Longissimus lumborum* stored for 7 d at 4 °C treated for 50 min with HIU: (**a**) HIU bath, lower area exposed to emitters; (**b**) HIU bath, upper unexposed area; (**c**) HIU probe, unexposed lower area; (**d**) HIU probe, upper area exposed to probe; (**e**) control without HIU, lower area; (**f**) control without HIU, upper area.

The scanning electron micrographs showed visual differences in the separation of the muscle fibers. Interestingly, a longer separation among muscle fibers was observed in the area exposed to the emitters of the sonic waves. Analysis of the micrographs showed that the highest interfibrillar separation occurred in the exposed area of the ultrasound-treated samples, regardless of the system (probe or bath). In an area of 10,000 µm^2^, 1813 µm^2^ of interfibrillar spaces were found on the surface of the muscle exposed to the ultrasonic emitters in the bath system (lower part of the muscle, Figure 3a), while on the unexposed or upper surface they were quantified only 705 µm^2^ of spaces among fibers (Figure 3b). Further, in the probe system an area of interfibrillar spaces of 1654 µm^2^ on the surface exposed to the probe (upper surface of the muscle, Figure 2d) and 965 µm^2^ on the unexposed surface were observed (lower surface of the muscle, Figure 3c). Finally, in the controls without HIU, an area of interfibrillar spaces of 871 µm2 was quantified on the lower surface and 992 µm^2^ on the upper surface of the muscle, after 7 d of storage at 4 °C (Figure 3e,f, respectively). The small spaces observed in the muscles without HIU (controls) were produced due to the effect of the aging process of the meat. The area of these spaces was similar to the unexposed areas of samples treated with HIU (bath and probe). The generation of spaces between fibers due to ultrasonication has been also observed by Carrillo-Lopez et al. [6]. They reported that HIU treatment in m. *Longissimus dorsi* dramatically increased interfibrillar areas when using a bath system (16, 28, and 90 W/cm^2^) for 20 and 40 min. However, no significant differences were found in shear force among samples treated with HIU and controls, which could be due to the use of thicker bags for packaging or to the interpretation of the results. In this regard, they observed a decrease in shear force from day 0 (immediately after the HIU) to 7 of storage. However, storage time and HIU intensity (0, 16, 29, and 90 W/cm^2^, and HIU time 20 and 40 min) could also be responsible for the lowest shear force. Furthermore, in a study by Carrillo-Lopez et al. [6], the samples were treated for 20 and 40 min with HIU (10 min and 20 min per side, respectively), while in the present study, the samples were not rotated, and the minimum treatment time to produce tenderization was 25 min. Therefore, it is hypothesized that the treatment time was not sufficient to produce changes in muscle tenderness. Microstructural studies of the muscle make it possible to explain the changes produced during tenderization of the meat. For this reason, the ultrasound treatments that presented large interfibrillar spaces (probe and bath, Figure 3a,d) had low values in shear force (Table 2). Treatment with HIU produces shock waves that damage the structure of the muscle, generating breakdown of cell membranes and physical weakening, which finally increases the tenderness of the meat [1]. Got et al. [19] reported that ultrasound treatment (2.6 MHz, 10 W/cm^2^, 2 × 15 s) before rigor in m. *Semimembranosus* (50 g samples) produced microstructural alterations in the region of the Z line and increased Ca release to the cytosol. However, there was no improvement in muscle tenderness after storage for 14 d, presumably due to the short treatment times.

## 3. Materials and Methods

### 3.1. Description of the Sample and Assignment of Treatments

Six loins of m. *Longissimus lumborum* were purchased from a commercial establishment. The loins were obtained from Beef Master heifers between 15 and 23 m old, with an average weight of 450 kg. The samples were received at −12 °C and were thawed for 24 h at 4 °C. The loins were cut transversely to obtain 2.5 cm thick slices. Visible fat and connective tissue were trimmed from the muscle, and the slices were immediately vacuum packed in 50 µm thick polyethylene bags (Koch Supplies Inc., Kansas City, MO, USA). Treatments were assigned according to a completely random factorial experimental design, resulting in six treatments and four replications. Two factors were established: the ultrasound factor (bath or probe) and the time factor (25 and 50 min). The slices were in direct contact with the ultrasonic waves from only one side, that is, the samples were not turned during the HIU application.

### 3.2. Ultrasonic Treatment

The vacuum-packed slices were treated under two ultrasound systems: Elmasonic^®^ S60H ultrasonic bath (Elmasonic S60H, Singen, Germany, 37 kHz, 550 W, 90 W/cm²) and Hielscher^®^ UP400St probe system (Berlin, Germany, 400 W, 24 kHz, continuous pulse and 100% amplitude). Distilled water was used as a diffusion medium, and the temperature was kept at 4 °C during the HIU treatment. The control consisted of slices of m. *Longissimus lumborum* not treated with HIU, kept at 4 °C to simulate the conditions of the ultrasonicated samples. The temperature was controlled with a portable immersion cooler immersed in the ultrasound systems (Julabo^®^ FT200, Württemberg, Germany). After ultrasonication, the samples were stored for 7 d at 4 °C. Once the storage period was completed, the physicochemical, microbiological, and microstructural determinations were carried out.

### 3.3. Physicochemical Evaluations

The color space measurement was determined according to the CIE L*a*b* color parameters of the CIE reference system (Commission Internationale Pour l’Eclarige, Vienna, Austria). The measurement was carried out with a Konica Minolta^®^ colorimeter (CR-400, Konica Minolta Sensing, Inc., Osaka, Japan; Illuminant C, 2° observer angle of measurement. Standard observer, C: Y = 94.2, x = 0.3130 and y = 0.3190. Aperture 8 mm) according to the methodology of the American Meat Science Association [39]. The meat was previously exposed to room conditions for 20 min to allow the oxygenation of myoglobin (blooming). 

The pH was evaluated with a digital meat pH meter (Sentron model 1001, Leek, The Netherlands) coupled to an immersion electrode. The readings were taken by introducing the electrode directly into the cut at a depth of 1.27 cm, according to the methodology of Honikel [40]. Readings were made in three areas of the muscle avoiding contact with fat and connective tissue.

The water holding capacity (WHC) was determined using the compression technique of Tsai and Ockerman [41]. For this procedure, 0.3 ± 0.1 g of meat (Ohaus Corporation model AV213, Parsippany, NJ, USA) was placed between two filter papers (Whatman No. 1), and constant weight of 10 kg was applied for 5 min using two methacrylate plates. The WHC was then calculated using the following expressions:

WHC (%) = 100 − Free water

Free water = ((final weight of filter paper-initial weight of filter paper)/sample) × 100

Shear force analysis was performed according to the methodology described by the AMSA [42]. Briefly, meat samples were cooked on electric plates (George Foreman^®^, Marshall, TX, USA) until the internal center reached 71 ± 0.1 °C. The temperature was monitored with a thermocouple probe. Cooked steaks were placed into plastic bags and refrigerated (1 °C) for 24 h before performing the shear force test. To take readings, a TA-XT-plus texturometer (Stable Micro Systems Ltd., Surrey, UK) was used. Eight to ten cylinders (1.27 cm ∅)/sample) were placed transversely and cut using a Warner Bratzler blade in a “V” shape (60° triangular opening) at a speed of 100 mm/min and a height of 30 mm. The maximum peak force is expressed in kgf.

### 3.4. Microstructural Analysis

For microstructural analysis, 5 mm diameter meat cylinders were obtained in the direction of the muscle fibers and were fixed in 2.5% glutaraldehyde in Sorensen’s phosphate buffer with a pH of 7.2. A post-fixation with 1% osmium tetroxide was carried out. Subsequently, the sections were dehydrated in a gradual series of ethanol (30–100%). The samples were critically dried with CO_2_ and mounted in aluminum sample holders using copper tape and then coated with a layer of gold (30 nm), to allow visualization of the surface in cross section. The prepared samples were observed in a JSM-6390 SEM scanning electron microscope (Jeol, Tokyo, Japan) operated with a voltage acceleration of 10 kV. The micrographs with magnifications of 200× were analyzed using the Image J software (Wayne Rasband, National Institute of Health, Bethesda, MD, USA), to characterize the fibers and interfibrillar spaces in triplicate in areas of 10,000 µm^2^.

### 3.5. Microbiological Analysis

Microbiological analyses included counts of total aerobic mesophilic bacteria, psychrophilic bacteria, coliform bacteria, and lactic acid bacteria (LAB). 1 mL of exudate was collected from each vacuum-packed sample and diluted from 1:10 to 1:1,000,000 using sterile diluent (MRD; peptone saline water prepared with 1.0 g/L peptone and 8.5 g/L sodium chloride, pH 7.0 ± 0.2). For samples that had a small amount of exudate, 1 g of sample was taken and placed in sterile bags to homogenize in a Stomacher^®^ 80 (Seward, AK, USA) for 60 s, using 9 mL of peptone water 0.1% (0.1 g/L peptones). Then, 100 µL of each dilution was inoculated into the specific medium described below using the plating technique [43]. For the determination of mesophilic and psychrophilic bacteria, nutrient agar (CM0325, Nutritive agar, Oxoid, Hampshire, UK) was used. Aerobic incubation was carried out at 35 °C ± 2 °C for 48 h and 168 h at 4 °C, respectively. For total coliforms, red-violet bile glucose agar (CM0485, Violet red bile glucose agar, Oxoid, Hampshire, UK) culture medium was used, incubating at 35 °C ± 2 °C for 48 h. One layer of 5 mL was added once the plates solidified to favor the growth of facultative coliforms, following the methodology of the FDA [44]. For the LAB count, De Man, Rogosa, and Sharpe agar culture medium (MRS, CM0361; Oxoid, Hampshire, UK) was used, and the incubation was carried out at 30 °C ± 2 °C for 120 h. To favor anaerobic conditions, a traditional method was used to deplete the oxygen in the medium.

### 3.6. Statistical Analysis

The results were analyzed using the statistical package SAS System 9.0 (Beijing, China). The reported values are means ± standard deviation. The variance analyses were performed under a completely randomized 3 × 2 factorial experimental design (HIU system factor in three levels and time factor in two levels, resulting in a total of six treatments). The difference of means was determined by Tukey’s tests (significance level *p* < 0.05). In the case of the microbiological variables, the CFU/mL were logarithmically transformed to log base 10. Pearson’s test was performed to detect correlations among dependent variables.

## 4. Conclusions

The color properties of m. *Longissimus lumborum* are not adversely affected by HIU. The lightness of the muscle in the bath system was significantly increased, compared with the control. Even though the muscle WHC decreased slightly in ultrasonicated muscle, and as the treatment time increased, a decrease in shear force was observed, without significant differences between ultrasound times. Electron microscopy studies corroborated the tenderness of the meat. While single-sided muscle exposure produced significant microstructural and physicochemical changes, both-sided exposure should provide higher effects. The total aerobic count and the total coliform bacteria counts were significantly increased in the sonicated samples. The ultrasound treatment also increased lactic acid bacterial counts in the bath system. An assisted technology should contribute to the efficient control of microorganisms, to take advantage of the benefits that the HIU treatment provides on tenderness and the improvement of the appearance of the meat.

## Figures and Tables

**Table 1:** Effect of the HIU system, HIU time, and the combination between them, on color parameters L*, a*, b*, tone (Hue), and chroma of bovine *Longissimus lumborum*.

Treatment	CIE L*a*b*				
HIU system	L*	a*	b*	Hue	Chroma
Control without HIU	37.9 ± 1.9 ^b^	19.1 ± 1.2 ^a^	8.5 ± 1.5 ^a^	23.5 ± 2.6 ^a^	20.9 ± 1.7 ^a^
HIU bath	40.6 ± 1.6 ^a^	20.3 ± 1.2 ^a^	8.6 ± 1.0 ^a^	23.0 ± 1.9 ^a^	22.0 ± 1.4 ^a^
HIU probe (100% amplitude)	39.8 ± 1.5 ^b^	20.1 ± 1.4 ^a^	8.8 ± 1.2 ^a^	23.5 ± 1.7 ^a^	21.9 ± 1.7 ^a^
HIU time (min)	L*	a*	b*	Hue	Chroma
25	39.5 ± 1.9 ^a^	19.5 ± 1.5 ^a^	8.4 ± 0.8 ^a^	23.0 ± 1.4 ^a^	21.2 ± 1.6 ^a^
50	39.3 ± 2.1 ^a^	20.1 ± 1.2 ^a^	8.9 ± 1.5 ^a^	23.6 ± 2.5 ^a^	22.0 ± 1.6 ^a^
HIU system*HIU time	L*	a*	b*	Hue	Chroma
Control					
25 min	38.1 ± 1.6 ^a^	18.3 ± 0.2 ^a^	7.9 ± 0.4 ^a^	22.8 ± 1.1 ^a^	19.9 ± 1.1 ^a^
50 min	37.7 ± 2.3 ^a^	19.8 ± 1.1 ^a^	9.0 ± 2.1 ^a^	24.1 ± 3.7 ^a^	21.8 ± 1.8 ^a^
Bath					
25 min	40.7 ± 1.3 ^a^	20.4 ± 1.4 ^a^	8.5 ± 0.9 ^a^	22.7 ± 1.3 ^a^	22.1 ± 1.6 ^a^
50 min	40.4 ± 2.1 ^a^	20.1 ± 1.1 ^a^	8.7 ± 1.3 ^a^	23.4 ± 2.5 ^a^	21.9 ± 1.4 ^a^
Probe					
25 min	39.6 ± 1.9 ^a^	19.9 ± 1.3 ^a^	8.7 ± 1.1 ^a^	23.6 ± 2.1 ^a^	21.7 ± 1.5 ^a^
50 min	39.9 ± 1.2 ^a^	20.3 ± 1.6 ^a^	8.8 ± 1.4 ^a^	23.4 ± 1.7 ^a^	22.2 ± 2.0 ^a^

^a,b^ Different letters within the same column indicate significant differences between treatments (*p* < 0.05).

**Table 2:** Effect of HIU system and HIU time factors on pH, water holding capacity (WHC,%), and shear force (kgf) of bovine *Longissimus lumborum*.

Treatment	Physicochemical Variables		
HIU system	pH	WHC (%)	Shear force (kgf)
Control without HIU	5.54 ± 0.07	57.73 ± 0.89 ^a^	2.41 ± 0.19 ^a^
HIU bath	5.33 ± 0.15	51.77 ± 4.32 ^b^	1.69 ± 0.22 ^b^
HIU probe (100% amplitude)	5.50 ± 0.16	54.1 ± 3.67 ^b^	1.82 ± 0.12 ^b^
HIU time (min)	pH	WHC (%)	Shear force (kgf)
25	5.41 ± 0.14	56.15 ± 3.31 ^a^	2.02 ± 0.44 ^a^
50	5.43 ± 0.16	52.90 ± 4.16 ^b^	1.92 ± 0.29 ^a^

^a,b^ Different letters within the same column indicate significant differences between treatments (*p* < 0.05).

**Table 3:** Pearson correlations and significances among dependent variables of beef *L. lumborum* without or with HIU application.

		a*	b*	C*	Hue	pH	WHC	SF	Meso	Psi	LAB	Coli
L*	Pearson C.	0.320	0.260	0.380	−0.297	−0.553	−0.457	−0.505	0.533	0.381	−0.076	0.462
	Sig.	0.195	0.297	0.120	0.231	0.017	0.056	0.033	0.023	0.119	0.766	0.054
a*	Pearson C.		0.120	0.985	−0.028	−0.069	−0.221	−0.567	0.505	0.568	−0.004	0.328
	Sig.		0.634	0.000	0.913	0.785	0.378	0.014	0.033	0.014	0.987	0.184
b*	Pearson C.			0.190	0.375	−0.165	0.061	−0.245	0.294	0.424	−0.033	0.049
	Sig.			0.450	0.125	0.514	0.810	0.328	0.237	0.079	0.896	0.847
C*	Pearson C.				0.009	−0.112	−0.181	−0.503	0.500	0.603	0.042	0.312
	Sig.				0.971	0.657	0.472	0.033	0.035	0.008	0.868	0.207
Hue	Pearson C.					0.153	0.049	0.088	−0.413	−0.186	0.076	−0.467
	Sig.					0.545	0.847	0.730	0.088	0.459	0.763	0.051
pH	Pearson C.						0.328	0.122	−0.409	−0.491	−0.428	−0.317
	Sig.						0.184	0.628	0.092	0.039	0.076	0.201
WHC	Pearson C.							0.670	−0.474	−0.050	−0.306	−0.679
	Sig.							0.002	0.047	0.843	0.217	0.002
SF	Pearson C.								−0.571	−0.332	0.245	−0.578
	Sig.								0.013	0.179	0.328	0.012
Meso	Pearson C.									0.745	0.169	0.845
	Sig.									0.000	0.503	0.000
Psi	Pearson C.										0.095	0.474
	Sig.										0.707	0.047
LAB	Pearson C.											0.258
	Sig.											0.301

WHC = water holding capacity. SF = shear force. Meso = mesophiles. Psy = psychrophiles. LAB = lactic acid bacteria. Coli = Coliform bacteria. Sig. = Significance of correlation.

**Table 4:** Effect of HIU system and HIU time on the count of mesophiles, psychrophiles, lactic acid bacteria, and coliforms of bovine *Longissimus lumborum* stored for 7 days at 4 °C.

Treatment	Microbiological Count (log_10_ CFU/mL)			
HIU system	Mesophiles	Psychrophiles	Coliforms	Lactic acid bacteria
Control without HIU	7.32 ± 0.17 ^b^	8.03 ± 0.23 ^a^	4.20 ± 0.19 ^b^	6.62 ± 0.31 ^a^
HIU bath	7.70 ± 0.23 ^a^	8.53 ± 0.29 ^a^	4.68 ± 0.21 ^a^	6.42 ± 1.25 ^a^
HIU probe (100% amplitude)	7.64 ± 0.31 ^a^	8.39 ± 0.30 ^a^	4.52 ± 0.23 ^a^	5.68 ± 1.18 ^b^
HIU time (min)	Mesophiles	Psychrophiles	Coliforms	Lactic acid bacteria
25	7.49 ± 0.20 ^a^	8.37 ± 0.30 ^a^	4.43 ± 0.17 ^a^	5.57 ± 0.99 ^b^
50	7.61 ± 0.35 ^a^	8.45 ± 0.26 ^a^	4.51 ± 0.37 ^a^	6.9 ± 0.54 ^a^

^a,b^ Different letters within the same column indicate significant differences between treatments (*p* < 0.05).

## Data Availability

The data presented in this study are available on request from the corresponding author.

## Sample Availability

Samples of microscopy studies are available from the authors.
